# What lies beneath the airway mucosal barrier? Throwing the spotlight on antigen‐presenting cell function in the lower respiratory tract

**DOI:** 10.1002/cti2.1158

**Published:** 2020-07-23

**Authors:** Saparna Pai, Visai Muruganandah, Andreas Kupz

**Affiliations:** ^1^ Centre for Molecular Therapeutics Australian Institute of Tropical Health and Medicine James Cook University Cairns QLD Australia

**Keywords:** airway mucosal barrier, airways, antigen‐presenting cells, lung, T cells

## Abstract

The global prevalence of respiratory infectious and inflammatory diseases remains a major public health concern. Prevention and management strategies have not kept pace with the increasing incidence of these diseases. The airway mucosa is the most common portal of entry for infectious and inflammatory agents. Therefore, significant benefits would be derived from a detailed understanding of how immune responses regulate the filigree of the airways. Here, the role of different antigen‐presenting cells (APC) in the lower airways and the mechanisms used by pathogens to modulate APC function during infectious disease is reviewed. Features of APC that are unique to the airways and the influence they have on uptake and presentation of antigen to T cells directly in the airways are discussed. Current information on the crucial role that airway APC play in regulating respiratory infection is summarised. We examine the clinical implications of APC dysregulation in the airways on asthma and tuberculosis, two chronic diseases that are the major cause of illness and death in the developed and developing world. A brief overview of emerging therapies that specifically target APC function in the airways is provided.

## Graphical abstract

We review the role of different antigen‐presenting cells (APC) in the lower airways and the mechanisms used by pathogens to modulate APC function during infectious disease. We discuss features of APC that are unique to the airways and their influence on uptake and presentation of antigen to T cells directly *in situ* in the airways. We summarize current information on the crucial role that airway APC play in regulating respiratory infection.

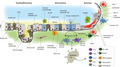

## Introduction

The prevalence of respiratory inflammatory and infectious diseases has increased significantly over the last few decades. The disappointing clinical efficacy of vaccines and drugs developed to prevent and treat respiratory diseases underscores our limited understanding of the immunoregulatory mechanisms of the airway microenvironment.^1^, ^2^ New immunological paradigms are urgently needed to drive vaccine development and drug discovery for respiratory diseases. Considering its significance, few advances have been made in our understanding of immunity to infection of the lower airways. The airways are exposed to a wide variety of inhaled antigens, and therefore, the induction of primary immunity to these antigens is tightly controlled by professional APC such as dendritic cells (DC) and macrophages (Table 1).^3^, ^4^ Various subsets of DC and macrophages in the airways act as ‘gate keepers’ to the lung and become activated soon after pathogen entry.^5^, ^6^ Once activated, they efficiently participate in phagocytosis, killing, antigen transport and co‐ordination of the innate and adaptive immune response, the caveat being that most antigens that reach the airway mucosal barrier (AMB) are harmless.^7^ Therefore, the discriminatory powers of the respiratory immune system are stretched to the limit as it must separate antigenic ‘noise’ from the rare pathogen signal. Once selected, it must regulate the immune response to these antigens to minimise collateral damage to the lung airways. The airways are therefore replete with mechanisms that prevent an inflammatory response, such as (1) setting the ‘default’ T‐cell response to a ‘tolerance’ mode (non‐inflammatory Th_2_ cell‐mediated immunity)^7^, ^8^; (2) induction of G_0_/G_1_ T‐cell cycle arrest^9^; (3) production of iNOS or IL‐10 by alveolar macrophages (AM)^10^; and (4) activation of FOXP3^+^ regulatory T cells (Treg),^8^, ^11^ all of which play a role in suppressing T‐cell activation at the AMB. Therefore, initiation of inflammation or an immune response requires a combination of events that override the ‘default’ inhibitory mechanisms at the AMB.^6^, ^7^ This review focuses on the immunological processes that regulate antigen uptake and presentation in the lower airways. Important areas that are discussed briefly owing to space limitations include immunoregulatory events in lymph nodes that drain the airways and Th_2_‐mediated inflammatory response leading to allergy/atopy *in vivo* in mice.

**Table 1:** Mouse surface markers of various APC subsets localised in the airways and lung

Cell type	Surface marker	Location	Function	References
Alveolar Macrophage	CD11c^hi^CD11b^−^F4/80^lo^MHC‐II^lo^CD206^hi^	Alveoli	Homeostasis, tissue remodelling, pathogen response	Hussell and Bell^6^ Lambrecht^3^ Jakubzick^22^
Interstitial Macrophage	Lyve‐1^hi^MHC‐II^lo^CD163^+^CD64^+^CD11b^+^CD206^+^	Peribronchial space	Immunosuppression	Schyns 2019^101^ Chakarov^25^
	Lyve‐1^lo^MHC‐II^hi^CD163^+^CD64^+^CD11b^+^CD206^−^	Alveolar interstitium	Antigen presentation	Schyns 2019^101^ Chakarov^25^
Airway cDC^++^	CD11c^hi^MHC‐II^hi^CD11b^+^CD4^−^CD8^−^CD205^+^	Airways	Antigen uptake Immunological tolerance	von Garnier^33^
Airway pDC^+^	CD11c^int^MHC‐II^lo^120G8^pos^	Airways	Host response to pathogens	von Garnier^33^
Alveolar DC	CD11c^+^CD11b^+^F4/80^lo^	Alveoli	Endocytosis	Guth^34^
*Tier 1 lung DC*	*Tier 2 lung DC*			
BATF3‐dependent cDC1	CD11c^+^CD11b^lo^CD103^+^ CD11c^+^CD11b^hi^CD103^−^	Lung		Guilliams^30^ Vroman^32^
IRF4‐dependent cDC2		Lung	Antigen uptake Migration to DLN	Guilliams^30^ Desch^5^
		Lung	Antigen uptake Migration to DLN	Guilliams^30^ Desch^5^
E2‐2‐dependent pDC	CD11c^hi^MHC‐II^int^B220^hi^Gr‐1^hi^	Lung	Immune regulation	Guilliams^30^ Desch^5^
Monocyte‐derived DC	CD11b^hi^CD64^+^ SIRPα^+^ CX3CR1^−^	Lung	Inflammation	Guilliams^15^

+pDC = Plasmacytoid DC; ++cDC = Conventional DC.

## Anatomical structure of the airways

**Figure 1:**
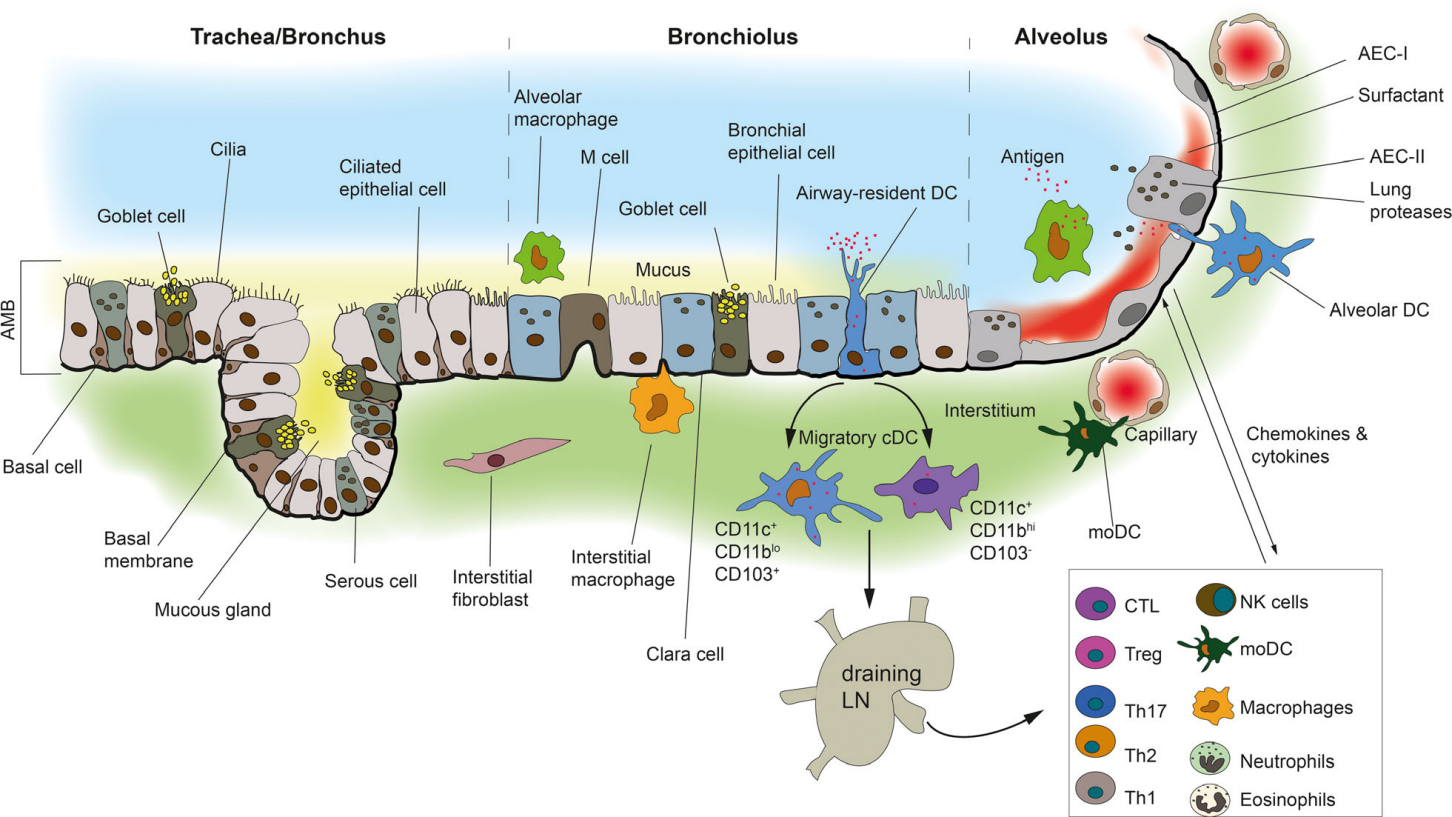
A schematic illustrating antigen‐presenting cell location and function at the AMB. The lower airways comprising the trachea, bronchus, bronchiolus and alveolus are shown. The tracheal wall is composed of ciliated pseudostratified epithelial cells containing goblet cells. The cilia propel mucus towards the pharynx. Mucus is formed mainly of mucins. As the trachea and the bronchus branch and become smaller, the mucosal epithelium thins and the pseudostratified epithelium changes to columnar and then cuboidal (as illustrated). The alveolar wall consists of AEC‐I and AEC‐II that is supported by a basement membrane and a sub‐epithelium containing a rich bed of blood vessels and immune cells. Epithelial barrier function is maintained by tight junctions and adherens junctions (not shown) that restrict epithelial permeability and immune cell migration. AEC‐II secrete a mixture of surfactants (shown in red). Surfactants are absent in the conducting airways which is covered by mucus (shown in yellow), an important distinguishing feature. The alveolar space is dominated by AM whereas the interstitium is dominated by IM. The AMB is populated by airway‐resident DC that are intraepithelial and uptake antigen. Migratory DC capture and ferry antigenic cargo from the airways to the DLN and present antigen to T cells. Secretion of cytokines and chemokines by airway epithelial cells recruits, mobilises and activates other key immune cells such as neutrophils, eosinophils, MoDC, NK cells, Treg and Th_2_ cells in the submucosa (shown in the box). All of these cells are involved in mediating an immune response against respiratory pathogens that have entered the airways and/or breached the AMB.

The airways can be divided into upper and lower tracts.^7^ The upper airway consists of the nose, nasal cavity, paranasal sinuses and the pharynx.^12^ The lower airway consists of the larynx, trachea, bronchi and bronchioles that end in tiny air sacs known as alveoli. The trachea divides to form the right and left bronchi that branch about 23 times to form a pattern called the bronchial tree (Figure 1). Passages smaller than 1 mm in diameter are called bronchioles. Combined, the airways of the lung have an approximate surface area of 70 m^2^ in humans.^7^ The main function of the upper airways is to warm, to humidify and to filter air. The lower airways consist of a conducting zone, which forms the respiratory passage for air, and a respiratory zone, which is the site of gas exchange.^12^ This zone is composed of the terminal bronchioles that feed into respiratory bronchioles. The respiratory bronchioles lead into winding alveolar ducts that terminate in clusters of alveoli.

The tracheal wall consists of several layers: mucosa, submucosa and adventitia overlying hyaline cartilage.^12^ The mucosa is composed of a ciliated pseudostratified epithelium containing goblet cells (detailed below) (Figure 1). Its cilia continually propel mucus towards the pharynx. Mucus is mainly formed of glycoproteins called mucins.^13^ MUC5AC and MUC5B are the most abundant mucins produced by the human epithelium besides membrane‐anchored mucins such as MUC1 and MUC4. Epidermal growth factor receptor (EGFR) plays a pivotal role in the synthesis and secretion of mucins.^13^ Mucins modulate the activity of alveolar macrophages (AM). The tissue composition of the bronchi is similar to the trachea.^12^ However, as the bronchi branch and become smaller, the following structural changes occur: (1) mucosal epithelium thins and changes from pseudostratified to columnar and then to cuboidal in the terminal bronchioles, and (2) mucus‐producing cells decrease in the bronchioles and the amount of smooth muscle in the wall increases, allowing bronchioles to resist air pressure (Figure 1). Unlike the tracheal wall, the alveolar wall consists of a single‐cell lining of respiratory epithelium composed of type‐I and type‐II alveolar epithelial cells (AEC) that is supported by a basement membrane and a sub‐epithelium containing a very rich vascular bed^14^ and immune cells such as DC and macrophages^15^ (Figure 1). The vascular bed is separated from the overlying respiratory epithelium by a mere 0.2 µm basement membrane, which allows for easy re‐oxygenation of blood.

## Alveolar macrophages

Alveolar macrophages are located almost exclusively in the alveolar space of the lung and play an instrumental role in lung immunity (Figure 1).^6^ They express CD11c^hi^CD11b^−^F4/80^lo^MHC‐II^lo^CD206^hi^ (Table 1). Under steady‐state conditions, AM constitute about 90% of the total leukocytes in the alveolar space and about 75–80% of the total macrophage population harvested from the bronchoalveolar lavage fluid (BALF) of rhesus macaques and humans.^3^, ^16^ AM are specialised macrophages that play a central role in homeostasis, tissue remodelling, immunosurveillance and host response to pathogens.^6^, ^17^ Attempts to classify AM into distinct subsets have led to a variety of descriptions,^18^ and it is widely accepted that they do not fit into any current macrophage classification.^6^ AM play an especially important role in the protection of the lower airways.^15^ AM‐ablated CD169‐DTR mice infected with PR8 influenza virus show increased virus load, severe airway inflammation, pulmonary edema and die from the disease.^19^ AM are ‘quiescent’ in the steady state, producing little inflammatory cytokines and displaying poor phagocytic activity.^6^ In this state, they actively suppress both alveolar and interstitial DC and T cells.^20^, ^21^ This is reinforced by the fact that DC recruitment and migration to draining lymph node (DLN) are boosted 20‐fold after AM depletion.^22^ Similarly, IL‐13‐dependent eosinophilic and Th_2_ inflammation is enhanced in mice depleted of AM using clodronate liposomes.^23^ However, DC suppression could be reversed by TLR stimulation which resulted in a potent inflammatory response.^24^ How do AM induce a switch between these two opposing functions of DC (suppression versus activation) following pathogen entry? AM adhering to AEC in the steady state induce the expression of αvβ6 integrin and TGF‐β, which suppress AM phagocytic activity.^18^, ^24^ Following TLR stimulation, TGF‐β is no longer produced by AEC, which results in AM switching to a pro‐inflammatory phenotype.

## Interstitial macrophages

Interstitial macrophages (IM) are heterogeneous macrophages that are conserved across tissues. They are involved in tissue homeostasis and inflammation.^25^ Two phenotypically distinct sub‐populations of IM, CD206^+^ and CD206^−/dim^ located in the peribronchial space and alveolar interstitium of the lung, respectively, have been identified (Table 1).^16^ CD206^+^ and CD206^−^ subsets largely overlap with Lyve‐1^hi^MHC‐II^lo^ and Lyve‐1^lo^MHC‐II^hi^ subsets described by Chakarov *et al*. Far fewer in number, the phenotypic expression of CD163, CD64 and CD11b by IM and localisation in the perivascular space resembles that of perivascular macrophages (PVM) that lie on the abluminal surface of post‐capillary venules and perform crucial activities at the blood–tissue interface.^16^ Detailed studies to assess whether IM have a similar function to that of PVM have not been conducted. However, fluorescent *Mycobacterium tuberculosis* (*Mtb*) reporter strains exhibited lower stress response and higher replication in AM compared to IM.^26^ Further, IM depletion increased bacterial burden suggesting that IM are superior at controlling bacterial growth.^25^ Difficulties associated with IM studies may be related to technical limitations of lung imaging, especially as bronchi and bronchioles are embedded deep in the lung tissue.^27^ Although lung explant imaging is a good alternative, the mucous layer and the ‘air–liquid interface’ are likely compromised in such *in situ* studies.^27^ In this context, DC have been reported to project trans‐epithelial extensions into the airway lumen; however, intravital studies were unable to observe this phenomenon.^28^ Therefore, it remains unclear whether inhaled particles are taken up by DC localised in the bronchioles or the alveoli.^29^ Regardless, there is evidence that IM contributes to AM replenishment as AM are long‐lived cells with negligible cell turnover.^16^ A rapid response to infection/injury requires accelerated recruitment of cells that have the plasticity to transform into AM, and IM fit this description. Further, stimulation of IM by IFN‐γ and LPS leads to superior expression of TNF‐α, suggesting that IM, more than AM, form the front line of mucosal defence in the alveoli.^16^

## Dendritic cells

DC play an important role at the AMB in inducing tolerance and determining the severity of inflammatory disease (Figure 1). Using a two‐tiered nomenclature suggested by Guilliams *et al*.,^30^, ^31^ mouse lung DC can be divided into three types under tier 1 – BATF3‐dependent cDC1 (conventional DC1), IRF4‐dependent cDC2 and E2‐2‐dependent pDC (plasmacytoid DC). A fourth type of DC generated under inflammatory conditions from monocytes is commonly known as monocyte‐derived DC (MoDC) (Table 1).^32^ Under tier 2, the cDC subset in the lung can be further differentiated based on the expression of CD103 or CD11b into CD11c^+^CD11b^lo^CD103^+^ and CD11c^+^CD11b^hi^CD103^−^. pDC in the lung are CD11c^hi^MHC‐II^int^B220^hi^Gr‐1^hi^. Mouse studies have generally used whole‐lung digests, and therefore, major differences between DC populations from the AMB versus parenchyma have not been appreciated. Mouse airways are dominated by CD11c^hi^MHC‐II^hi^CD11b^+^CD4^−^CD8^−^CD205^+^ DC, and subsets typically seen in lymphoid tissues such as CD8α^+^ DC and CD4^−^CD8^−^ DC are absent.^33^ Additionally, a small population of CD11c^int^MHC‐II^lo^120G8^pos^ pDC is also detected in the airways. On the other hand, DC in the alveoli are CD11c^+^CD11b^+^F4/80^lo^.^34^ Flow cytometric surveys have identified several airway‐ and alveolar‐resident phagocytes in the BALF of healthy humans. Besides AM, CD14^+^ DC and CD14^−^ DC (macrophage‐like) and Langerin^+^, BDCA1^+^CD14^+^ and BDCA1^+^CD14^−^ DC (DC‐like) have been identified.^35^ In the Itgax‐YFP or CX_3_CR1‐GFP mice in which cDC express YFP and monocytes/macrophages express GFP, respectively, cDC are located near the large airways, whereas monocytes and AM are localised in the alveolar space.^32^ MoDC are located at the interface of blood vessels and airways in MacBlue mice, whereas pDC are located in the alveolar interstitium. A balance between cDC and pDC in the lung is crucial for the outcome of pulmonary infection as Flt3L‐mediated DC expansion is compromised in pDC‐depleted mice.^36^ Further, depletion studies revealed that pDC‐induced Treg expansion via Sema4a‐mediated pathway protects against severe asthma.^37^ pDC activation also alleviates airway hyperreactivity by increasing the apoptotic rate of ILC2 through IFN‐α production.^38^ Immunostaining of the rat trachea has revealed that only 20% of total airway mucosal DC population are found within the airway epithelium while the remaining 80% lie beneath the epithelium.^39^ Intraepithelial DC have endocytic activity, whereas the subepithelial cells do not. Freshly isolated airway DC are functionally immature, MHC‐II^lo^, endocytosis^hi^, mixed lymphocyte reaction^lo^, IL‐10^hi^ and stimulate Th_2_ responses.^40^ They retain a somewhat ‘immature’ phenotype, even after moving to the DLN, and this is likely to be critical for the development of immune tolerance. Exposure of DC to thymic stromal lymphopoietin (TSLP), TGF‐β and vascular endothelial growth factor (VEGF) or even interaction with AM may promote this tolerogenic phenotype. Alteration in the tolerogenic phenotype of airway DC leads to exaggerated Th_2_ responses to inhaled antigens and allergic airway inflammation.^41^ In line with this, conditional depletion of airway DC by treatment of thymidine kinase‐transgenic mice with the antiviral drug ganciclovir resulted in a significant decrease in the number of bronchoalveolar CD4^+^, CD8^+^ T and B lymphocytes and eosinophilic airway inflammation.^42^ However, as mentioned, this topic is beyond the scope of this review.

## Epithelial cells

Epithelial cells of the AMB are being increasingly implicated in infectious disease pathogenesis, although their role in asthma/allergy has been known for some time.^43^ The respiratory epithelium composed of AEC‐I and AEC‐II is a physical barrier involved in modulating innate immunity and rapid clearance of environmental agents (Figure 1).^14^ AEC‐II, which constitute about 7% of the total alveolar surface, secrete cytokines and chemokines and express MHC‐II constitutively on their cell surface, a phenotype rarely exhibited by non‐professional APC.^44^, ^45^ AEC‐II secrete a mixture of surfactants known as surfactant protein A (SP‐A), SP‐B, SP‐C and SP‐D named after their chronologic order of discovery.^46^ Surfactant is formed of lipid membranes (90%) and proteins (10%) that reduce surface tension in the alveoli.^13^ SP‐A and SP‐D are water‐soluble and belong to the family of collectins (collagenase‐like lectins). SP‐B and SP‐C occur in significantly smaller amounts than SP‐A. Collectins play a major role in lung defence as shown by their capacity to inactivate respiratory virus and gram‐negative bacteria. They modulate AM response by binding to SIRPα, TLR4 and TLR2 receptors, enhance phagocytosis of bacteria by acting as opsonins, and regulate inflammation by modulating neutrophil chemotaxis.^13^ Proteases produced by AEC‐II and neutrophils degrade surfactant proteins and modulate immune cells whereas anti‐proteases produced by pathogens such as *Pseudomonas aeruginosa* (Pa) elastase and Pa protease IV counter this effect. AEC‐II‐derived factors may also play an important role in promoting inflammation, regulating DC function and controlling bacterial growth.^44^, ^45^ AEC can express a variety of adhesion molecules and soluble mediators such as TGF‐β, prostaglandins, nitric oxide, TSLP and IL‐10 through which they are likely to modulate DC function within the airway. *In vitro* studies have also shown that AEC‐II present mycobacterial proteins to MHC‐II‐restricted CD4^+^ T cells.^45^ Why would AEC‐II present antigen to T cells when other professional APC such as AM and DC reside in the AMB? The answer may lie in the necessity to elicit a rapid recall response to certain pathogens, such as *Mycobacteria*. For example, the memory T‐cell response (especially the CD8^+^ subtype, which are less reliant on T‐cell receptor (TCR) signalling and APC co‐stimulation) is crucial for protection against *Mtb* and may be stimulated by non‐professional APC.^47^ Of interest, AEC‐II does not express detectable levels of CD80 and CD86 and induces tolerance to environmental antigens in naïve T cells.^44^, ^45^ Thus, AEC‐II may be a crucial player that affects the balance between tolerance and inflammation in the AMB.

## Antigen uptake in the airways

DC and AM are both strategically positioned in the airways for antigen uptake (Figure 1). They are the predominant phagocytic populations of the lung, in the steady state and disease.^5^ Studies have shown that both DC and macrophages extend processes into the airways for antigen uptake, similar to DC in dermal micro‐vessels and intestinal tissue.^5^ Interlinked sheets of tracheal CD103^+^ DC line the mucosal surface.^48^ These DC associate tightly with the epithelium via the integrin CD103 and are rarely harvested in the BALF. Experiments using immunohistochemical staining have positioned them on the basal side of the epithelium wherein they express tight junction proteins such as Claudin‐1, Claudin‐7 and Zonulin.^49^ These molecules enable DC protrusions to capture antigen from the airway lumen, as shown by DC reaching across the airspace to engulf fluorescent particles. After which, they traffic to the DLN to elicit T‐cell responses.

Two subsets of migratory cDC, CD11c^+^CD11b^lo^CD103^+^ and CD11c^+^CD11b^hi^CD103^−^, have been identified in the conducting airways of healthy rats with a turnover rate of 2–3 days.^5^ They capture and ferry antigenic cargo from the airways to the DLN and present antigen to T cells (Figure 1). However, in order for this to occur, antigens must get past physical barriers such as the mucociliary escalator, surfactants and highly phagocytic AM. In this context, DC have been reported to acquire antigen in the airways once a complete antigenic saturation of AM (approx. 10^9^ organisms) occurs.^50^ Once activated, AM can engulf up to 10 bacteria each before there is a spillover to the DC compartment.^50^ This may be one of many mechanisms AM use for suppressing T‐cell responses – limiting the access of DC to pulmonary pathogens. This is supported by studies on AM‐depleted rodents in which lung DC show enhanced APC function and T cells acquire a pro‐inflammatory phenotype.^20^ Taken together, AM are more likely to suppress antigen presentation function rather than directly suppress T cells.^22^ Certain intracellular pathogens have developed mechanisms to exploit site‐specific immunosuppression by AM to their advantage. The immunosuppressive activity of AM may therefore contribute to disease severity by enabling pathogens to replicate before they are overcome by immune cells. For example, while AM express high levels of iNOS in *Mtb*‐infected patients and kill *Mtb* directly,^51^ they also suppress T‐cell activation.^52^ Furthermore, in the early stages of *Mtb* infection in mice, the bacteria inhaled into the respiratory airways via aerosol are taken up by AM, which are unable to migrate to the DLN, resulting in delayed trafficking of antigen and T‐cell activation.^53^ Meanwhile, *Mtb* burden in the lung increases by 10 000–100 000‐fold in mice leaving the ensuing T‐cell response unable to eradicate the pathogen.^54^ However, T‐cell responses are efficiently induced when mycobacterial antigens of the early secreted antigenic target 6 kda (ESAT‐6) protein family are engineered to circumvent AM and targeted into specialised DC subsets via CD11b or CD11c β2‐integrins.

## Antigen presentation in the airways

After antigen acquisition, CD103^+^ DC traffic to the DLN at a lower frequency than CD11b^hi^ DC.^55^, ^56^ This limitation of CD103^+^ DC can be offset by their capacity to ferry larger quantities of antigen per cell, suggesting differential capacities in these two subsets for acquiring and presenting antigen. Another major difference is the exclusive capacity of CD103^+^ DC to acquire, transport and cross‐present apoptotic cell‐associated antigen to CD8^+^ T cells in the DLN.^56^ Thus, the primary role of trafficking cellular, particulate and soluble antigen from the airways to the DLN lies with the migratory subsets of CD103^+^ and CD11b^hi^ DC, but not plasmacytoid DC, both during steady state and infection. Once DC reach DLN, they present antigen to CD4^+^ and CD8^+^ T cells (Figure 1).^57^ While CD11b^hi^ DC present antigen via MHC‐II promoting CD4^+^ T‐cell response, CD103^+^ DC cross‐present soluble antigen via MHC‐I to CD8^+^ T cells.^57^ In contrast, AM take up particulate matter delivered intranasally but do not migrate to DLN.^22^ Therefore, they are not considered to have a role in antigen presentation in lymphoid tissue. After stimulation by DC, naive T cells undergo multiple rounds of cell division in the DLN and emigrate back to the site of infection in the lung where they can further interact with viral antigen‐displaying inflammatory DC.^58^ This second round of T‐cell interaction with DC in the infected lung tissue is crucial because *in vivo* depletion of lung DC leads to defective viral clearance. In this context, CD4^+^CD25^+^FOXP3^+^LAG3^+^CTLA4^+^CD45RC^+^ Treg cells play a central role in controlling airway DC function and allergen sensitisation in rats.^59^ Treg maintain immune homeostasis in the lung by suppressing inflammation in both steady state and infectious disease.^7^ In respiratory syncytial virus (RSV)‐infected mice, Treg depletion leads to delayed viral clearance and excessive pulmonary inflammation.^60^ The role of pDC in assisting Treg homeostasis is understudied; however a recent report points to an important role for this interaction in the regulation of Klebsiella‐pneumonia progression.^61^

It is important to distinguish antigen presentation in DLN from that occurring locally in the lung or at the AMB. In a landmark study, Saini *et al*.^62^ showed that local DC antigen presentation occurs in the lung in early stages of respiratory infection. Mice infected with a *Mtb* variant (deleted for the gene PE_PGRS47) showed enhanced MHC class II‐restricted antigen presentation by DC in all organs *in vivo*. Therefore, the antigenic load at the site of infection is critical for the timely arrival of CD8^+^ T cells into the airways, and antigen availability is also a crucial factor for CD8^+^ T‐cell activation in the lung.^63^ CD4^+^ T cells show increased IFN‐γ production when administration of synthetic peptides led to increased antigen presentation in lung lesions.^64^ Further, an increased number of migration‐arrested, IFN‐γ‐producing effector T cells home into areas of the lung that have abundant antigen. One of the earliest studies used OVA‐coated fluorescent latex beads to track the sites and timing of antigen uptake and presentation within the airways.^65^ Using this approach, beads were detected in lung APC as early as 2 h after intranasal injection. The data show that antigen‐loaded APC can traffic from the airways to the lung almost immediately. This is followed by greater numbers of transferred OVA‐specific Th_1_ but not Th_2_ or naïve cells localising with bead^+^ APC. Based on these observations, the authors concluded that productive antigen/APC/T‐cell interactions can take place in the lung even before the antigen has reached the DLN.^66^ Such early encounters of T effector cells with antigen may be especially important for the development of a robust secondary (recall) immune response. Additionally, studies have shown that intranasally delivered antigens were taken up and presented to antigen‐specific T cells by CD11c^hi^ APC that remained within lung tissues and did not migrate to secondary lymphoid organs.^66^

The time‐consuming process of trafficking antigen to the DLN becomes redundant under certain condition such as asthma, which begins within 8 h of antigen inhalation.^67^ During influenza infection for example, trans‐presentation of IL‐15 directly in the lung by MHC class I‐antigen‐loaded DC has the same effect as antigen presentation to CD8^+^ T cells in the DLN.^68^ Seminal studies have suggested that antigen‐bearing DC can fast‐track antigen presentation in the airways by directly presenting antigen to T cells *in situ*.^29^ However, the conundrum is that only 3% of airway‐resident DC project processes between the epithelial cells, and even those never extend past the epithelium into the airspace. On the other hand, even though alveolar DC actively extend and retract dendrites along the AMB to capture antigen‐loaded microspheres, antigen‐specific T cells preferentially interact with airway‐resident DC, rather than alveolar DC.^29^ These data suggest that direct antigen presentation at the AMB, if and when it occurs, is a highly regulated process in the steady state. It appears that DC are granted access to antigen only in the most distal lung compartment thus restricting immune response to the most invasive pathogen. Airways and alveoli are therefore treated as distinct sites by our immune system, and the differential capacity for T‐cell activation by APC may be a consequence of such a compartmentalisation.^7^ Dysregulation of these tightly controlled processes in certain microenvironments therefore invariably leads to disease.

## Lung niches

Spatially, ‘compartmentalised niches’ such as inducible bronchus‐associated lymphoid tissue (iBALT) and nasal‐associated lymphoid tissue (NALT) also play a role in the immunity of the respiratory tract that is distinct from that in lymphoid or peripheral tissues.^69^, ^70^ Influenza‐specific CD4^+^ and CD8^+^ resident memory T cells are expanded and maintained long term in iBALT, which are essentially sites of regeneration after tissue injury.^71^ As T cells are replaced every 10 days in the airways, they must undergo continual recruitment from the circulation for their maintenance.^72^ How do these circulatory T cells transition to a sessile form in the airways? Recent data show that encounter with cognate antigen is a key event for the conversion of circulating to resident T cells in the lung; however, the site or cells involved have not been identified.^69^ Whether compartmentalised niches serve as a reservoir for antigen in the airway is unknown. It is clear however that spatial niches are important for protection against secondary infections by respiratory pathogens. Unlike antigen‐dependent T‐cell activation in the lung, tissue‐resident memory (known as T_RM_) CD8^+^ T cells develop independently of cognate antigen recognition and persist in the airways to provide long‐term protection.^73^ Given the intensity of antigenic exposure in the airways, this seems logical as T‐cell activation events require strict control at this site. This may also explain why lung and airway T_RM_ show distinct differences in their repertoire and differential ability to respond to antigen. Immune regulation may be achieved among other mechanisms by locking memory T cells into G_0_/G_1_ phase of the cell cycle.

## Therapeutic targets of APC function – A clinical perspective

### Asthma

Asthma is a chronic, inflammatory disease of the airways that accounts for a significant portion of morbidity and mortality worldwide. It is characterised by airway hyper‐responsiveness, mucous cell hypertrophy, overproduction of mucous and airway remodelling. A highly heterogeneous disease asthma can be broadly categorised into four subsets: eosinophilic, neutrophilic, mixed granulocytic and paucigranulocytic. DC have been implicated in the development of asthma in both murine models and human studies.^32^ There is a growing pool of evidence demonstrating DC capacity to skew the allergen priming of the pulmonary immune system towards a Th_2_ or Th_17_ phenotype resulting in eosinophilic or neutrophilic asthma, respectively. Impaired inducible costimulatory ligand (ICOL) expression on DC has been shown to promote the development of aberrant Th_2_ responses in patients with asthma.^74^ Likewise, upregulation of programmed death ligand 2 (PD‐L2) has been implicated with Th_2_ priming. Blockade of PD‐L2 with antibodies *in vitro* resulted in decreased cytokine production, highlighting a possible DC target for therapy.^75^

Another therapeutic approach may be by manipulating the airway milieu. TSLP which is expressed by activated lung epithelial cells promotes Th_2_ responses by activating plasmacytoid and thymic stromal lymphopoietin protein receptor (TSLPR)^+^ DC.^76^ TSLP has been implicated in asthma, with specific polymorphisms being associated with an increased susceptibility to developing the disease.^77^ Tezepelumab, an anti‐TSLP human monoclonal antibody, has shown efficacy in inhibiting both early and late asthma responses, as well as blood eosinophilia and clinical parameters.^78^ This highlights the importance of investigating the TSLP pathway further as a target for asthma therapy. Aside from its role in preventing IgE binding, omalizumab, another monoclonal antibody used in the treatment of asthma, is capable of suppressing Th_2_ responses perpetuated by DC.^79^ The activation of peroxisome proliferator‐activated receptor (PPAR)‐mediated pathways in DC appears to circumvent Th_2_ airway inflammation through the induction of IL‐10. Rosiglitazone, a PPAR agonist and antihyperglycaemic drug, has shown some capacity to suppress inflammation in a subset of asthmatic patients.^80^ Furthermore, Lee *et al*.^81^ have shown that rosiglitazone may be able to prevent airway remodelling during chronic asthma. Similarly, metformin has been associated with decreased airway eosinophilic inflammation in mice^82^ and decreased asthma exacerbations in humans.^2^ Taken together, these studies and many others demonstrate the inextricable role DC play in skewing airway inflammation to cause an eosinophilic phenotype in the context of asthma.

Interestingly, AM appears to remain relatively stable in asthma, and therefore, they have not attracted as much attention. Nonetheless, impaired macrophage function has been identified as a key player in asthma. AM isolated from asthmatic patients have a reduced capacity to phagocytose bacteria and foreign substances such as carbon particulate matter.^83^ This may allow for the altered microbiome observed in asthmatic patients to contribute to bacterial‐induced exacerbations. Similarly, AM isolated from patient with non‐eosinophilic asthma show impaired efferocytic capacity (ability to clear dead or dying cells), allowing for the accumulation of cellular debris and neutrophils in the airways, which potentially contributes to the chronic inflammatory state.^84^ Therefore, restoration of the phagocytic function of AM may serve as an interesting target for novel therapies. One possible avenue may be the inhalation of GM‐CSF, which has been previously used in clinical studies to treat cystic fibrosis patients with *M. abscessus* infection.^85^

### Tuberculosis

Tuberculosis (TB) continues to be a major global health concern. In particular, the emergence of multidrug‐resistant (MDR) and extensively drug‐resistant (XDR) *Mtb* strains poses an increasing burden on health systems in many regions of the world.^86^ Although new anti‐tuberculosis drugs, such as pretomanid and bedaquiline, have shortened the lengthy treatment regimens, particularly of MDR‐TB and XDR‐TB,^87^, ^88^ drug toxicity and side effects will likely contribute to poor compliance and, hence, future resistance development. In addition, it remains uncertain whether any of the currently trialled TB vaccine candidates will show superiority over BCG and prevent pulmonary tuberculosis in adolescents and adults.

While considerable research efforts have been directed towards the development of new anti‐TB drugs and vaccines, the equally important area of adjunctive host‐directed therapies (HDTs) has attracted significantly less attention.^89^ HDTs enhance the immune system's intrinsic capacity to deal with cancerous cells or to neutralise pathogenic bacteria, while limiting tissue pathology. In TB, most of the current research on HDT is focused on modulating the permissiveness of macrophages to *Mtb* infection, but no current anti‐TB drug specifically targets the intracellular AM niche.^90^ Due to experimental limitations in human lung, most of our current knowledge about the distinct functional role of individual APC subsets, including AM, is derived from animal studies. Nevertheless, there is increasing evidence that human AM may also be amenable to targeted interventions, and experiments using human lung explants have provided critical new insights into early responses of AM after contact with *Mtb*.^91^ Much like in asthma, AM from patients infected with TB have diminished phagocytic capacity and furthermore have a decreased ability to activate intracellular bacterial killing pathways. This is due to *Mtb* modulating phagolysosome fusion through its ESX secretion systems, allowing for intracellular persistence and replication.^92^ Several existing drugs, such as metformin, imatinib, aspirin, pioglitazone and alisporivir, have been shown to interfere with recognition, uptake and/or killing of *Mtb* by macrophages. The implicated host pathways in this interference include the modulation of autophagy, phagosomal maturation, lipid and sugar metabolism and induction of necrosis, among others (elegantly reviewed by Machelart *et al.* ^90^). To what extent licensed anti‐TB drugs' anti‐mycobacterial activity is augmented by host‐targeting mechanisms remains underexplored.

Another exciting aspect of HDT in TB is centred on targeting myeloid‐derived suppressor cells (MDSCs).^93^ MDSCs are a heterogeneous group of myeloid‐derived monocytic and polymorphonuclear phagocytes with strong inhibitory potential on innate and adaptive immune responses.^94^ Due to their inhibitory potential on T cells, MDSCs have gained significant attention in the cancer field, and it is now appreciated that MDSCs also impair *Mtb* control. In susceptible mouse strains, MDSCs also increase in the lung in parallel with increasing TB burden,^95^ and in humans, MDSCs are increased in frequency in active TB.^96^ As such, MDSCs constitute an attractive HDT target in TB. Pharmacologically, several aspects of MDSC‐centred HDT have been proposed, including reversal of the inhibitory MDSC phenotype, depletion of MDSCs and inhibition of MDSC recruitment.^93^ Trialled compounds for these strategies include etanercept, gefitinib, imatinib, anti‐IL‐6R, anti‐VEGF, IDO inhibitors, COX‐2 inhibitors and calprotection.^93^

Additionally, the potential to use DC‐therapy as an HDT in TB has also gained momentum. Satake and colleagues recently demonstrated that MoDC from TB patients could successfully be polarised into type‐1 DC, and when loaded with *Mtb* antigens induced substantially stronger *Mtb*‐specific T‐cell responses, superior activation of NK cells and enhanced suppression of Treg compared to standard DC.^97^ From a future HDT perspective, it can be envisaged that lung‐resident DC could be targeted directly *in vivo* towards polarisation into a type‐1 phenotype, or alternatively, *ex vivo* polarised and antigen‐loaded DC could be reinfused into the lung of TB patients.

In order to progress many of these potential HDTs towards clinical TB application, a better understanding of how to most efficiently deliver compounds into the respiratory tract is required. While nanoparticles and liposomes have been trialled to deliver drugs directly into AM for some time,^98^ biodegradable glucose polymer‐based nanoparticle approaches may prove to be more efficient in reducing side effects and enhancing drug delivery to the site of infection and into specific APC cell subsets.^99^ Collectively, the development of host‐directed immunotherapies that harness and optimise intrinsic antibacterial immune mechanisms, especially in macrophages and DC, will likely complement current antibiotic protocols to deliver more personalised TB treatments in the future.

## Future perspectives

The confluence of cell biology and imaging has improved the sensitivity and spatio‐temporal resolution of events that take place in the airways and lung during infectious disease. As a consequence, fascinating insights into the interaction of immune cells, especially APC and pathogens, are certain to emerge. There are multiple APC‐mediated processes that regulate T cells at the AMB: (1) poor antigen presentation by AM; (2) tolerogenic phenotype of DC and limited ability to uptake antigen; and (3) non‐professional APC such as AEC‐II having a tolerogenic effect on naïve T cells. These biological processes have rarely been visualised in real time in the lung, and adopting new technologies will immensely benefit such studies. Exploration of the ‘gut–lung axis’, wherein the microbiome of the gut is believed to influence susceptibility to pulmonary disease, is also becoming a key stream of infectious diseases research. The gut microbiome has been shown to affect lung susceptibility to viral, fungal and bacterial pathogens because of the common developmental origins the digestive and respiratory systems share. Consequently, gut–lung crosstalk is expected to become a fertile area of study in the future. A relatively unexplored domain for possible novel therapeutics is naturally occurring products derived from fauna and flora. Such products have shown great promise in suppressing the pathology and clinical signs associated with inflammatory diseases and are expected to shape future areas of study.^100^

## Conflict of interest

The authors declare no conflict of interest.

## Author contributions

SP wrote the manuscript. VM contributed to clinical perspectives. AK revised the manuscript and provided critical input.
